# Dynamical Complexity Fingerprints of Occupation-Dependent Brain Functional Networks in Professional Seafarers

**DOI:** 10.3389/fnins.2022.830808

**Published:** 2022-03-18

**Authors:** Hongjie Yan, Huijun Wu, Yanyan Chen, Yang Yang, Min Xu, Weiming Zeng, Jian Zhang, Chunqi Chang, Nizhuan Wang

**Affiliations:** ^1^Department of Neurology, Affiliated Lianyungang Hospital of Xuzhou Medical University, Lianyungang, China; ^2^School of Biomedical Engineering, Health Science Center, Shenzhen University, Shenzhen, China; ^3^Key Laboratory of Behavioral Science, Center for Brain Science and Learning Difficulties, Institute of Psychology, Chinese Academy of Sciences, Beijing, China; ^4^Center for Brain Disorders and Cognitive Science, Shenzhen University, Shenzhen, China; ^5^Lab of Digital Image and Intelligent Computation, Shanghai Maritime University, Shanghai, China; ^6^School of Pharmacy, Health Science Center, Shenzhen University, Shenzhen, China; ^7^Peng Cheng Laboratory, Shenzhen, China; ^8^School of Biomedical Engineering, ShanghaiTech University, Shanghai, China

**Keywords:** brain entropy, dynamical complexity, efficiency, graph theory, occupational neuroplasticity, seafarer, small-worldness

## Abstract

The complexity derived from resting-state functional magnetic resonance imaging (rs-fMRI) data has been applied for exploring cognitive states and occupational neuroplasticity. However, there is little information about the influence of occupational factors on dynamic complexity and topological properties of the connectivity networks. In this paper, we proposed a novel dynamical brain complexity analysis (DBCA) framework to explore the changes in dynamical complexity of brain activity at the voxel level and complexity topology for professional seafarers caused by long-term working experience. The proposed DBCA is made up of dynamical brain entropy mapping analysis and complex network analysis based on brain entropy sequences, which generate the dynamical complexity of local brain areas and the topological complexity across brain areas, respectively. First, the transient complexity of voxel-wise brain map was calculated; compared with non-seafarers, seafarers showed decreased dynamic entropy values in the cerebellum and increased values in the left fusiform gyrus (BA20). Further, the complex network analysis based on brain entropy sequences revealed small-worldness in terms of topological complexity in both seafarers and non-seafarers, indicating that it is an inherent attribute of human the brain. In addition, seafarers showed a higher average path length and lower average clustering coefficient than non-seafarers, suggesting that the information processing ability is reduced in seafarers. Moreover, the reduction in efficiency of seafarers suggests that they have a less efficient processing network. To sum up, the proposed DBCA is effective for exploring the dynamic complexity changes in voxel-wise activity and region-wise connectivity, showing that occupational experience can reshape seafarers’ dynamic brain complexity fingerprints.

## Introduction

### Entropy-Based Complexity of Brain Activity

Strong brain plasticity appears in the first few years of life, but various factors throughout adulthood still affect the morphology of brain structure and function. Occupation is a key factor that shapes our brain, which is closely related to our daily life, and exerts a subtle and persistent influence on brain plasticity changes (Wang N. et al., 2017, 2018; Dottori et al., 2020; Wei et al., 2020; Wu et al., 2020). Some studies have shown that the complexity of brain organization is an effective biomarker that reflects the individual’s brain health level (Goldberger et al., 2002), aging (Sokunbi et al., 2011), cardiovascular diseases (Richman and Moorman, 2000), the neural effects of drug use (Ferenets et al., 2007), etc. Recently, research shows that entropy, a useful measure of brain complexity, can be used to investigate the impact of diseases (Sokunbi et al., 2014; Wang B. et al., 2017; Lin et al., 2019) and aging (Sokunbi et al., 2015) on the human brain, implying that it is feasible to use entropy to explore the brain’s complexity changes caused by occupational factors (Wang N. et al., 2017, 2018; Wu et al., 2020). Since neural activity can be periodic, chaotic, or random (Gu et al., 2014; Yilmaz et al., 2016), the complexity level of brain signals will be different (Wang et al., 2014; DiNuzzo et al., 2017), where entropy can measure the signal randomness and predictability of a random process. Lower entropy indicates a low complex and more regular signal or system. Moreover, as the activity captured by the BOLD signal in brain areas over time is dynamical (Deco et al., 2008; Calhoun et al., 2014; Preti et al., 2017; Vidaurre et al., 2017; Lurie et al., 2020), the dynamical complexity is potential to characterize occupational neuroplasticity and is the main focus of this study.

### Complex Network-Based Complexity of Brain Connectivity

Characterizing the human brain as a complex system can help to interpret the evolution of brain structure and function (Richman et al., 2004; Bullmore and Sporns, 2009; Avena-Koenigsberger et al., 2018). With the development of resting-state functional magnetic resonance imaging technology (rs-fMRI), it can provide the feasible solution for complex networks to assess the functional topology among different brain areas over time (Onias et al., 2014). Lahaye et al. (2003) indicated that the non-linear information of interactions between BOLD signals plays a key role in identifying important connections. The key difference is that the non-linear technique produced networks with scale-free degree distributions (van den Heuvel et al., 2008; Pritchard et al., 2014), and the network hubs dominated information processing in the brain network (Mears and Pollard, 2016). Regarding the construction of functional networks based on complex networks, the linear correlations between brain regions are usually used like Pearson correlation coefficient (PCC) (Achard et al., 2006; Hayasaka and Laurienti, 2010). For example, Jung et al. (2013) used the methods of voxel-based morphometry (VBM), linear functional connection, and graph theory analysis to explore the changes in brain structure and function associated with professional Baduk players. Furthermore, in an rs-fMRI study of Chinese chess players, Duan et al. (2014) also pointed out that compared to non-professional subjects, professional chess players showed stronger functional connectivity between learning and memory areas, as well as stronger small-worldness topology. However, human brain information processing functions exist at multiple levels of interaction and non-linear behavior, which may be affected by electrical, chemical, and physical components controlled by thresholds and saturation phenomena (Abásolo et al., 2006; Sokunbi et al., 2014). Moreover, studies have shown that after Gaussian smoothing of fMRI data, there are significant but few non-linear characteristics (Hartman et al., 2011; Hlinka et al., 2011). For example, Xie et al. (2008) found significant non-linear features in fMRI data when the non-linear methods were used. Additionally, previous studies showed the non-linear functional connectivity-based features were beneficial for the classification of schizophrenia (Su et al., 2013). Together, the complex network based on brain entropy sequences measured by sample entropy is an effective united model to perform the linear, non-linear, and scale-free analysis for brain functional networks.

### Our Study

Based on the aforementioned discussion, brain activity has dynamical transitions and linear and non-linear features. Meanwhile, the entropy-based complexity and complex network-based complexity can be viewed as a typical measure of the human brain system. Further, the sample entropy is sensitive to both linear and non-linear analysis and is robust to the length of signal, which provides the potential strategy for revealing the complexity analysis of linear, non-linear, dynamics, and network topology.

Thus, in the current study, we hypothesize that professional seafarers might differ from non-seafarers on: (1) the dynamic complexity changes of brain functional plasticity, especially areas associated with motor coordination; (2) the global topological properties of a dynamical entropy-based complex network, according to previous evidence that long-term expertise training caused functional neuroplasticity changes. Using dynamic brain entropy mapping analysis and brain-entropy-based complex network analysis on the rs-fMRI dataset, we expect to detect changes in dynamic complexity and topological complexity features of brain activity driven by occupations in seafarers, for proposing a dynamic brain complexity analysis framework (DBCA) to decode brain activity by combing which was made up of dynamic brain entropy mapping analysis and brain-entropy-based complex network analysis. Moreover, we applied the proposed DBCA framework to explore the dynamic complexity and topological complexity changes of brain activity caused by occupation between the professional seafarers and non-seafarers based on the rs-fMRI dataset.

The remainder of this paper is organized as follows: The Material and Methods section related to the theory of DBCA framework, data acquisition, and processing will be presented first, followed by the experimental results section. Finally, the analysis is presented together with interpretations, discussion, and conclusions related to DBCA and complexity fingerprints of occupation-dependent brain functional networks in professional seafarers.

## Materials and Methods

### Data Acquisition and Preprocessing

All MRI data from subjects of 20 seafarers (all male, mean age of 49 years, right handed) and 20 education level-, gender-, and age-matched healthy control subjects (all male, mean age of 51 years, right handed) were collected from the Shanghai Key Laboratory of Magnetic Resonance. All participants were informed about the purpose of the study and signed a written consent form according to procedures approved by the IRB of East China Normal University (ECNU). For all subjects, no history of mental illness or neurological diseases were reported, and all seafarers have more than 10 years of sailing experience, and they work and live under strict militarized management on the sea. On the contrary, the recruited control subjects were from the land work position of university campus security guard without long-term sailing experience. For more details, please refer to Wang N. et al. (2017, 2018).

The scanning parameters were listed as follows: GE 3.0 Tesla with gradient echo EPI, slice number = 36, covering the whole brain area; sensitivity acceleration factor = 2.0, time of repetition (TR) = 2.0 s, scanning resolution = 80*80, in-plane resolution = 3.75 mm × 3.75 mm, slice thickness = 4 mm, time points = 160.

All data preprocessing was performed by DPARSF^1^, which is based on the SPM^2^ and REST^3^ toolbox. The preprocessing steps for rs-fMRI data of each subject in this study are as follows: (1) slice timing with the 39th slice as reference slice; (2) realignment; (3) normalization by EPI template (resampling voxel size = 3 mm × 3 mm × 3 mm); (4) spatial smooth by a Gaussian kernel with FWHM = 8mm.

### Dynamical Brain Complexity Analysis Framework

**FIGURE 1:**
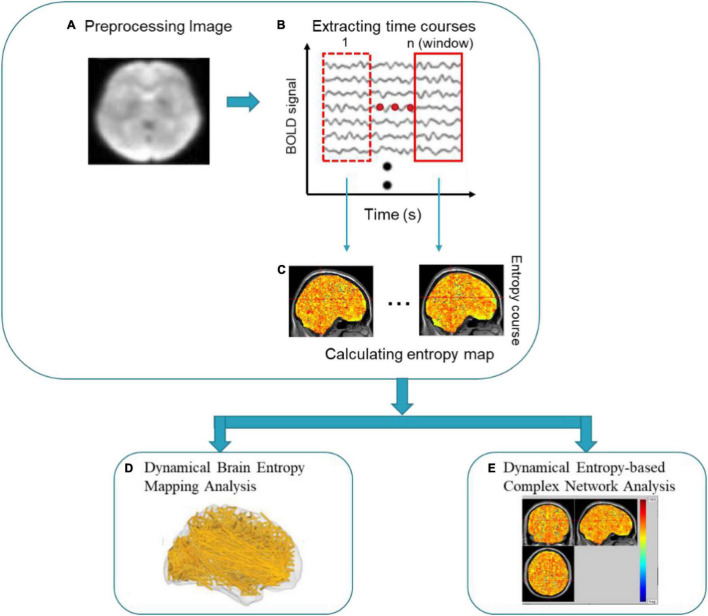
Framework of dynamical brain complexity analysis (DBCA).

Previous studies have shown the dynamic remodeling property in brain function during the task or task-free condition reflected by complexity (Sporns, 2011; Cole et al., 2013; Calhoun et al., 2014; Zalesky et al., 2014; Braun et al., 2015; Preti et al., 2017; Vidaurre et al., 2017; Shi et al., 2019; Fu et al., 2020; Lurie et al., 2020). In this section, the DBCA framework based on sample entropy (*SampEn*) was proposed for capturing the dynamical complexity, which is applied to measure the occupation-dependent complexity fingerprints of the professional seafarers. The DBCA framework was depicted in Figure 1, which was made up of dynamic brain entropy mapping analysis and brain-entropy-based complex network analysis, generating the dynamic complexity mapping of local brain areas and the topological complexity features of connectivity across the brain areas, respectively. Specifically, we firstly extracted BOLD signals from rs-fMRI data by the sliding window method and establish dynamic sample entropy mappings of seafarers and non-seafarers, and then analyze and discuss the results of the dynamic entropy maps. Further, we applied a *SampEn*-based approach to establish functional connections in complex network analysis between brain regions of the dynamical entropy map sequences, where the *SampEn* measurement was proved to be sensitive to both non-linear and linear correlations (Pritchard et al., 2014).

### Formation of Dynamical Brain Entropy Mapping Analysis

The sliding window method is used to slide a certain window length on the time axis in a given step to extract the BOLD signal within the window, thereby obtaining the time-varying BOLD signal in the brain region (Lindquist et al., 2014; Hindriks et al., 2016; Jia et al., 2017; Vergara et al., 2019). Studies have shown that the effect of data length (*N*) on *SampEn* is small when the data length gradually decreases from *N* = 128 to *N* = 85, accompanying the gradual increase of its noise (Sokunbi, 2014; Wang et al., 2014). Therefore, in order to achieve a relatively higher signal-to-noise ratio, in this paper, we chose a window length of 70 (80, 90) TRs, about 140–180 s. Then, the transient complexity map of brain activities was calculated by *SampEn* of the sliding window data. Finally, the brain imaging data of the seafarers and non-seafarers were analyzed based on the dynamic time series of the brain entropy maps (see Figure 1). The detailed procedures and formulation of dynamic brain entropy mapping analysis were presented as follows:

(1) With regard to the preprocessed fMRI images, using the sliding window method, the time window with a given window length of 70 (80, 90) TRs slides in steps of 1 TR, resulting in 81 (71, 61) windows. As mentioned earlier, the fMRI image sequence is represented by *X*_1_, *X*_2_, …., *X*_*N*_, and the given window length is *Len*, the fMRI image sequence contained in the *i*th window is *X*_*i*_, *X*_*i*+1_, …, *X*_*i*+*Len*−1_; a total of *M* = *N* − *Len* + 1 time windows are obtained.

(2) In a given *i*th time window, with the seafarers and non-seafarers data described earlier, a brain entropy map based on *SampEn* was constructed for each of the subjects. For the calculation of *SampEn*, we set the parameter*m* (initial embedding dimension) equal to 2 based on preliminary investigations (Sokunbi, 2014). Following Wang et al. (2014), we set the parameter *r* (similarity criterion) equal to 0.3 of the average of standard deviation (SD) of each pair of the time series. In brief, let*u* and *v* represent two time series: *u* = [*u*(1), *u*(2), …, *u*(*N*)] and *v* = [*v*(1), *v*(2), …, *v*(*N*)], where *N* is the number of data points in each series. With *m* and *r* fixed (as defined earlier), we form two vector sequences *x*_*m*_ and *y*_*m*_: *x*_*m*_(*i*) = [*u*(*i*), *u*(*i* + 1), …, *u*(*i* + *m* − 1)] and *y*_*m*_(*i*) = [*v*(*i*), *v*(*i* + 1), …, *v*(*i* + *m* − 1)]. Then, for each *i* ≤ *N* − *m* + 1, we define


(1) $$B_{i}^{m}\,\left(r\right)={\left(N-m+1\right)}^{-1}\,\left\{j\leq N-m|d\,\left(x_{m}\,\left(i\right),y_{m}\,\left(j\right)\right)<r\right\}$$

Where *j* ranges from 1 to *N* − *m*(*j* ≠ *i*), and *d*(*x*_*m*_ (*i*), *y*_*m*_(*j*)) is the distance function. $B_{i}^{m}\,\left(r\right)$ is the distance within *r*. Then, let


(2) $$B^{m}\,\left(r\right)={\left(\text{N-m}\right)}^{-1}\,\sum_{i=1}^{N-m}B_{i}^{m}\,\left(r\right)$$

Similarly, we extend to *m + 1*, and define $A_{i}^{m}\,\left(r\right)$and *A^m^* (*r*)as


(3) $$A_{i}^{m}\,\left(r\right)={\left(N-m+1\right)}^{-1}\,\left\{j\leq N-m|d\,\left(x_{m+1}\,\left(i\right),y_{m+1}\,\left(j\right)\right)<r\right\}$$

(4) $$A^{m}\,\left(r\right)={\left(\text{N-m}\right)}^{-1}\,\sum_{i=1}^{N-m}A_{i}^{m}\,\left(r\right)$$

Finally, we can calculate *SampEn* as


(5) $$S\,a\,m\,p\,E\,n\,\left(m,r,N\right)=-l\,n\,\left[A^{m}\,\left(r\right)/B^{m}\,\left(r\right)\right]$$

The formula 5 is used to calculate the complexity of the BOLD signal in several sliding windows, resulting in the sample entropy value *E*_*i*_ of the BOLD signal within the *ith* window.

(3) Calculate the average dynamic entropy maps (Mean-BEN) and the standard deviation of dynamic entropy maps (Std-BEN) of the dynamic entropy maps for each subject in the seafarer group and non-seafarer group, respectively.

(4) After the Mean-BEN and the Std-BEN were calculated, the REST software (Song et al., 2011) was used to perform a two-sample *t*-test between two groups of mean and standard entropy maps with AlphaSim correction (individual voxel *p* = 0.01, FWHM = 8 mm, and 1,000 iterations). The results were displayed and located by MRIcro software^4^ and wfu_pickatlas software (Maldjian et al., 2003, 2004), respectively.

**FIGURE 2:**
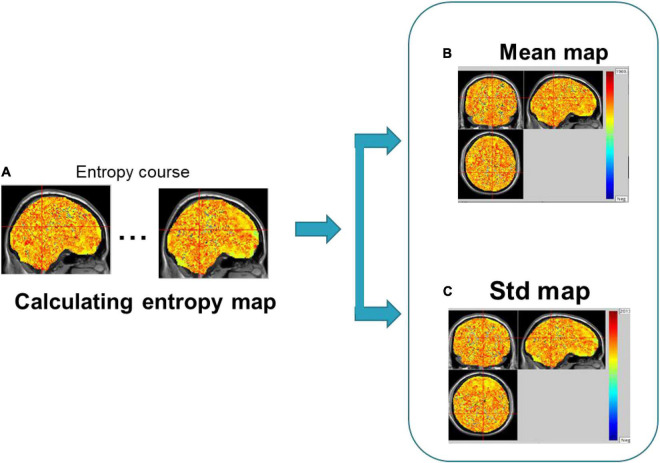
Framework of dynamical brain entropy mapping analysis.

The framework of dynamical brain entropy mapping analysis is displayed in Figure 2.

### Dynamical Entropy Based Complex Network Analysis

**FIGURE 3:**
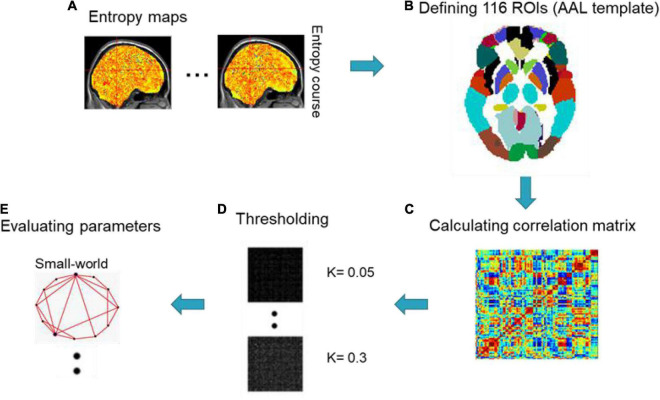
Framework of dynamical entropy-based complex network analysis.

The connection patterns in the human brain have been characterized as various networks, from microscopic neurons (Yu et al., 2008; Bonifazi et al., 2009) to macroscopic whole-brain analysis (Achard et al., 2006; Hagmann et al., 2008; Hayasaka and Laurienti, 2010; Yao et al., 2013; Wang N. et al., 2015; Wang et al., 2016; Shi et al., 2018, 2021; Yang et al., 2019, 2020; Ou et al., 2020; Nie et al., 2021). In order to unveil the topological properties of functional networks caused by occupation between the seafarers and non-seafarers, we used different sliding window lengths to obtain dynamic brain entropy sequences and then applied Pearson correlation coefficients (PCC) and the Automated Anatomical Labeling (AAL) template (Tzourio-Mazoyer et al., 2002) to calculate the correlation of the whole brain interval, so as to calculate the whole brain network connectivity. After creating a correlation network, based on proportional thresholds (*K*), we calculated two interesting topological parameters based on complex network analysis for the seafarers and non-seafarers: small-worldness and efficiency (Wang J. et al., 2015). The framework of dynamical entropy-based complex network analysis is displayed in Figure 3.

#### Small-Worldness

Small-worldness is an important concept that characterizes the principles of organization. These principles are reflected in various social operations, business activities, and biological networks (Latora and Marchiori, 2001). The small-worldness is an important attribute of brain functional network, which describes the separation or integration of network functions.

A network with small worldness should meet two conditions: normalized clustering coefficient $\gamma =\frac{C_{p}}{C_{r\,a\,n\,d}}>1$ and normalized characteristic path length λ = *L*_*p*_/*L*_*rand*_ ≈ 1, where *C*_*p*_ and *L*_*p*_ are the average clustering coefficient and average path length on all nodes in the target network, respectively (Watts and Strogatz, 1998); *C*_*rand*_ and *L*_*rand*_ are the average clustering coefficient and average characteristic path length of the random network, respectively (Maslov and Sneppen, 2002). Generally, random networks have smaller clustering coefficients and shorter characteristic paths due to their randomness. Lower *L*_*p*_ indicates higher communication efficiency between the entire brain area (Kaiser and Hilgetag, 2006; Rubinov and Sporns, 2010). In order to avoid the influence of another network, the normalized *C*_*p*_ and *L*_*p*_ are calculated, respectively. Compared with random networks, small-world networks have similar *L*_*p*_ and higher *C*_*p*_, so small-world networks can be quantified as $S=\frac{\gamma }{\lambda }>1$.

#### Efficiency

Efficiency is an index that describes the brain network from the perspective of biological and functional information flow within the brain (Rubinov and Sporns, 2010). The efficiency of the whole brain network and the regional one can be evaluated.

Global efficiency: the shorter the shortest path length *L*_*i*_ of a node, the faster the information transfer between the node and other nodes, which means the higher the global efficiency *E*_*glob*_ of the node (Rubinov and Sporns, 2010). We define it as


(6) $$E_{g\,l\,o\,b}=\frac{1}{n\,\left(n-1\right)}\,\sum_{i\in N}\sum_{j\neq i\in N}\frac{1}{d_{i\,j}}$$

where *d*_*ij*_ denotes the shortest path length between nodes *i* and *j*.

Local efficiency: Local efficiency is the same attribute as global efficiency, but the calculation of local efficiency is performed in the neighborhood of a node in the network (Rubinov and Sporns, 2010). The formulation can be expressed as


(7) $$E_{l\,o\,c}=\frac{1}{n}\,\sum_{i\in N}E_{l\,o\,c}\,\left(G_{i}\right)$$

where *E*_*loc*_(*G*_*i*_) is the local efficiency of *G*_*i*_.

## Experimental Results

### Dynamical Entropy Maps Between Seafarers and Non-seafarers

**FIGURE 4:**
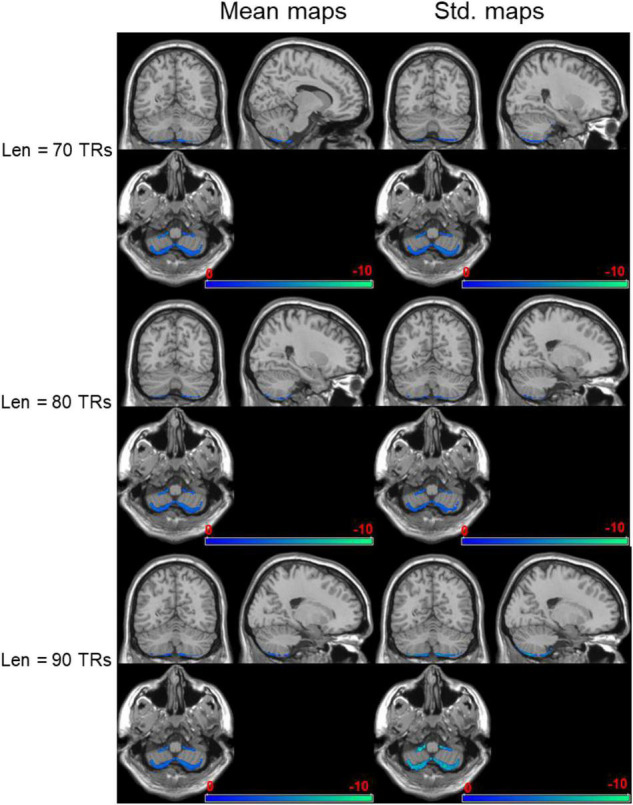
Negatively activated brain regions regarding dynamical brain entropy maps (seafarers’ entropy value < non-seafarers’ entropy value; AlphaSim correction, *p* < 0.05).

The negatively activated brain areas using dynamical brain entropy mapping analysis under different window length (*Len* = 70, 80, 90 TRs) shown in Figure 4 indicates that the dynamical brain entropy value of seafarers is lower than that of the control group. The activated area is mainly concentrated in the cerebellum, especially the bilateral Cerebelum_8 brain area in the AAL brain atlas. The MNI coordinates and the corresponding areas are recorded in Tables 1–3, respectively.

**TABLE 1:** MNI coordinates of negatively activated brain regions and related brain regions (seafarers’ brain entropy value < non-seafarers’ brain entropy value, *Len* = 70 TRs, AlphaSim correction, *p* < 0.05 with cluster size > 207 voxels).

	Area	MNI (*x, y, z*)	Peak intensity	AAL atlas
Mean-BEN	Cerebellum posterior lobe	(10, −76, −52)	−7.1814	Cerebelum_9_R/Cerebelum_8_R
	Inferior semi-lunar lobule	(−20, −40, −57)	−5.5496	Cerebelum_8_L
Std-BEN	Cerebellum posterior lobe	(10, −76, −52)	−6.8113	Cerebelum_9_R/Cerebelum_8_R
	Inferior semi-lunar lobule	(−20, −40, −57)	−5.705	Cerebelum_8_L

**TABLE 2:** MNI coordinates of negatively activated brain regions and related brain regions (seafarers’ brain entropy value < non-seafarers’ brain entropy value, *Len* = 80 TRs, AlphaSim correction, *p* < 0.05 with cluster size > 210 voxels).

	Area	MNI (*x, y, z*)	Peak intensity	AAL atlas
Mean-BEN	Cerebellum posterior lobe	(10, −76, −52)	−6.9932	Cerebelum_9_R/Cerebelum_8_R
	Inferior semi-lunar lobule	(−20, −40, −57)	−5.3811	Cerebelum_8_L
Std-BEN	Cerebellum posterior lobe	(10, −76, −52)	−5.7403	Cerebelum_9_R/Cerebelum_8_R
	Inferior semi-lunar lobule	(−20, −40, −57)	−5.1791	Cerebelum_8_L

**TABLE 3:** MNI coordinates of negatively activated brain regions and related brain regions (seafarers’ brain entropy value < non-seafarers’ brain entropy value, Len = 90 TRs, AlphaSim correction, *p* < 0.05 with cluster size > 202 voxels).

	Area	MNI (*x, y, z*)	Peak intensity	AAL atlas
Mean-BEN	Cerebellum posterior lobe	(10, −76, −52)	−7.2451	Cerebelum_9_R/Cerebelum_8_R
	Inferior semi-lunar lobule	(−20, −40, −57)	−5.3966	Cerebelum_8_L
Std-BEN	Cerebellum posterior lobe	(10, −76, −52)	−11.8983	Cerebelum_9_R/Cerebelum_8_R
	Inferior semi-lunar lobule	(−20, −40, −57)	−7.1306	Cerebelum_8_L

**FIGURE 5:**
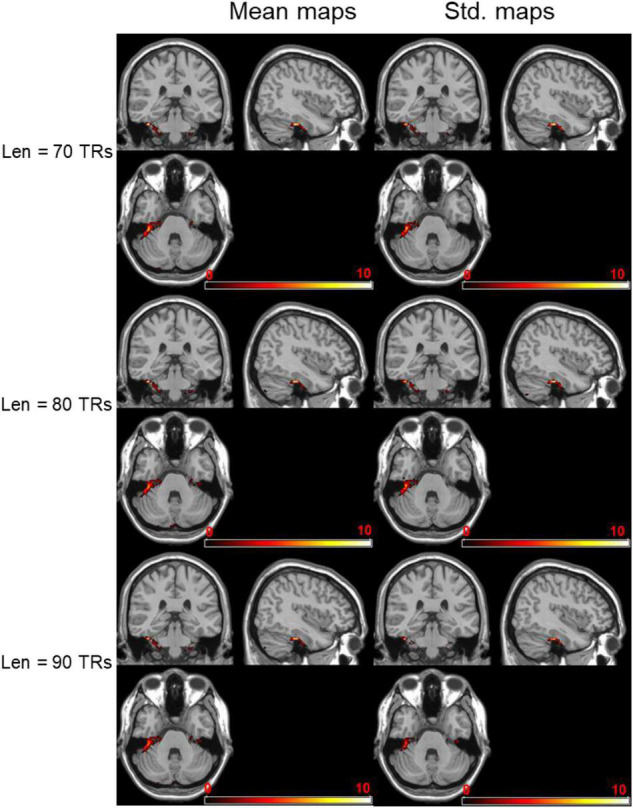
Positively activation brain regions regarding dynamic brain entropy maps (seafarers’ entropy value > non-seafarers’ entropy value; AlphaSim correction, *p* < 0.05).

The positively activated brain areas using dynamical brain entropy mapping analysis under different window length (*Len* = 70, 80, and 90 TRs) shown in Figure 5 indicates that the seafarer’s dynamic brain entropy value exceeds the expected value. The activation area is mainly concentrated in the fusiform gyrus, especially the brain area Brodmann area 20 (BA20), which involves the activated brain area, and the corresponding MNI coordinates are recorded in Tables 4–6, respectively.

**TABLE 4:** MNI coordinates of positively activated brain regions and related brain regions (seafarers’ brain entropy value > non-seafarers’ brain entropy value, Len = 70 TRs, AlphaSim correction, *p* < 0.05 with cluster size > 207 voxels).

	Area	MNI (*x, y, z*)	Peak intensity	AAL atlas (Brodmann area)
Mean-BEN	Fusiform gyrus	(−42, −32, −32)	15.6783	Fusiform_L (BA20)
Std-BEN	Fusiform gyrus	(−42, −32, −32)	16.4746	Fusiform_L (BA20)

**TABLE 5:** MNI coordinates of positively activated brain regions and related brain regions (seafarers’ brain entropy value > non-seafarers’ brain entropy value, Len = 80 TRs, AlphaSim correction, *p* < 0.05 with cluster size > 210 voxels).

	Area	MNI (*x, y, z*)	Peak intensity	AAL atlas (Brodmann area)
Mean-BEN	Fusiform gyrus	(−42, −32, −32)	14.1881	Fusiform_L (BA20)
Std-BEN	Fusiform gyrus	(−42, −32, −32)	14.4102	Fusiform_L (BA20)

**TABLE 6:** MNI coordinates of positively activated brain regions and related brain regions (seafarers’ brain entropy value > non-seafarers’ brain entropy value, Len = 90 TRs, AlphaSim correction, *p* < 0.05 with cluster size > 202 voxels).

	Area	MNI (*x, y, z*)	Peak intensity	AAL atlas (Brodmann area)
Mean-BEN	Fusiform gyrus	(−42, −32, −32)	12.4208	Fusiform_L (BA20)
Std-BEN	Fusiform gyrus	(−42, −32, −32)	7.2726	Fusiform_L (BA20)

### Dynamical Entropy-Based Complex Network Analysis Between Seafarers and Non-seafarers

**FIGURE 6:**
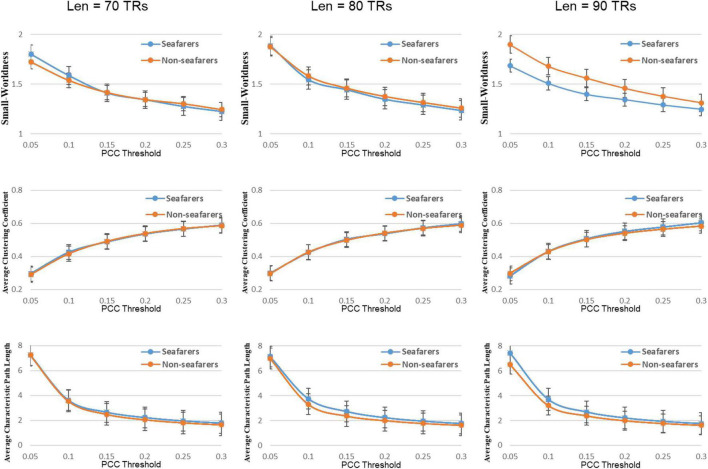
Average values of small world attributes and their parameters of the seafarers and non-seafarers based on brain entropy time series under different window lengths and different thresholds.

**FIGURE 7:**
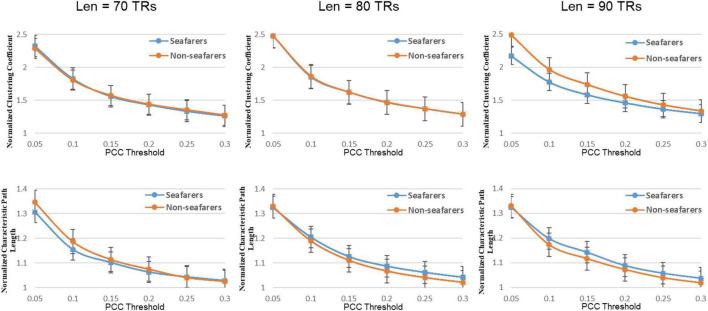
The average normalized path length and average normalized clustering coefficient of the seafarers and non-seafarers based on brain entropy time series under different window lengths and different thresholds.

**FIGURE 8:**
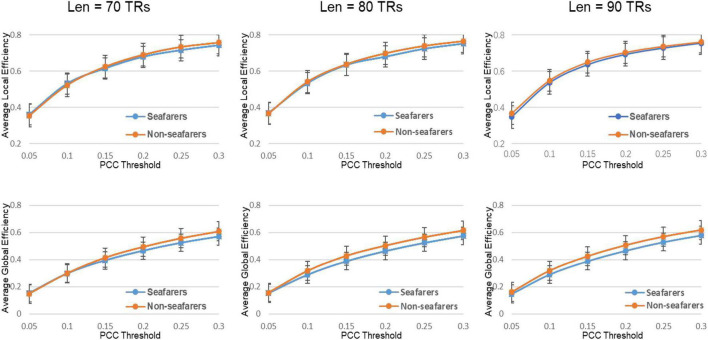
The average value of the efficiency attribute and its parameters of the seafarers and non-seafarers based on brain entropy time series under different window lengths and different thresholds.

First, the average and variance of small world attributes (i.e., small world, feature path length, and clustering coefficient) and network efficiency (i.e., global efficiency and local efficiency) within the range of 0.05 ≤ *K* ≤ 0.3 (step size = 0.05) under different sliding window lengths were calculated for the seafarers and non-seafarers, and the results are shown in Figures 6–8, respectively. Second, the two groups compared across the proportional thresholds (*K*) by means of two-way analysis of variance (ANOVA), the details are listed in Tables 7–9, respectively.

**TABLE 7:** The mean of all network parameters for seafarers and non-seafarers at Len = 70 TRs are presented, as well as the results of two-way ANOVA (*p*-value).

Properties	Parameters	Groups	Thresholds						
			0.05	0.1	0.15	0.2	0.25	0.3	*p*-value
Small-world	*C*_*p*_	Seafarers	0.29	0.43	0.49	0.54	0.57	0.59	0.3702
		Non-	0.29	0.42	0.49	0.54	0.57	0.59	
	γ	Seafarers	2.33	1.83	1.56	1.43	1.33	1.26	0.8918
		Non-	2.29	1.81	1.57	1.44	1.35	1.27	
	*L*_*p*_	Seafarers	7.27	3.64	2.67	2.24	1.97	1.80	**0.005**
		Non-	7.25	3.56	2.49	2.06	1.81	1.65	
	λ	Seafarers	1.31	1.15	1.10	1.06	1.04	1.03	0.1024
		Non-	1.35	1.19	1.11	1.07	1.04	1.02	
	*S*	Seafarers	1.80	1.59	1.41	1.35	1.28	1.23	0.4718
		Non-	1.73	1.54	1.42	1.35	1.30	1.25	
Network efficiency	*E*_*glob*_	Seafarers	0.15	0.30	0.39	0.47	0.53	0.57	**0.0352**
		Non-	0.15	0.30	0.42	0.50	0.56	0.61	
	*E*_*loc*_	Seafarers	0.36	0.53	0.62	0.68	0.72	0.74	0.2325
		Non-	0.35	0.52	0.63	0.69	0.74	0.76	

*Values in bold indicate statistically significant differences.*

**TABLE 8:** The mean of all network parameters for seafarers and non-seafarers at Len = 80 TRs are presented, as well as the results of two-way ANOVA (*p*-value).

Properties	Parameters	Groups	Thresholds						
			0.05	0.1	0.15	0.2	0.25	0.3	*p*-value
Small-world	*C*_*p*_	Seafarers	0.30	0.42	0.50	0.54	0.57	0.60	0.1694
		Non-	0.30	0.43	0.50	0.54	0.57	0.59	
	γ	Seafarers	2.48	1.85	1.62	1.46	1.37	1.29	0.6618
		Non-	2.48	1.86	1.62	1.47	1.37	1.29	
	*L*_*p*_	Seafarers	7.14	3.76	2.73	2.24	1.96	1.77	**0.0038**
		Non-	6.97	3.31	2.36	2.00	1.77	1.63	
	λ	Seafarers	1.33	1.21	1.13	1.09	1.06	1.04	**0.0125**
		Non-	1.33	1.19	1.11	1.07	1.04	1.02	
	*S*	Seafarers	1.89	1.55	1.45	1.35	1.29	1.24	**0.0336**
		Non-	1.88	1.58	1.46	1.38	1.32	1.26	
Network efficiency	*E*_*glob*_	Seafarers	0.15	0.29	0.39	0.47	0.53	0.58	**0.0049**
		Non-	0.16	0.32	0.43	0.50	0.57	0.62	
	*E*_*loc*_	Seafarers	0.37	0.53	0.64	0.68	0.73	0.75	**0.0331**
		Non-	0.37	0.54	0.64	0.70	0.74	0.77	

*Values in bold indicate statistically significant differences.*

**TABLE 9:** The mean of all network parameters for seafarers and non-seafarers at Len = 90 TRs are presented, as well as the results of two-way ANOVA (*p*-value).

Properties	Parameters	Groups	Thresholds						
			0.05	0.1	0.15	0.2	0.25	0.3	*p*-value
Small-world	*C*_*p*_	Seafarers	0.28	0.43	0.51	0.55	0.58	0.60	0.2785
		Non-	0.30	0.43	0.50	0.54	0.56	0.58	
	γ	Seafarers	2.17	1.78	1.58	1.46	1.36	1.29	**0.0174**
		Non-	2.49	1.97	1.74	1.56	1.43	1.33	
	*L*_*p*_	Seafarers	7.42	3.70	2.68	2.20	1.92	1.75	**0.0296**
		Non-	6.49	3.20	2.38	1.99	1.76	1.62	
	λ	Seafarers	1.31	1.20	1.14	1.09	1.06	1.04	**0.0160**
		Non-	1.33	1.17	1.12	1.07	1.04	1.02	
	*S*	Seafarers	1.69	1.51	1.40	1.34	1.29	1.25	**0.0022**
		Non-	1.90	1.68	1.56	1.46	1.38	1.31	
Network efficiency	*E*_*glob*_	Seafarers	0.15	0.29	0.39	0.47	0.53	0.58	**0.0005**
		Non-	0.16	0.32	0.43	0.51	0.57	0.62	
	*E*_*loc*_	Seafarers	0.35	0.54	0.64	0.69	0.73	0.76	**0.0009**
		Non-	0.37	0.55	0.65	0.70	0.74	0.76	

*Values in bold indicate statistically significant differences.*

## Discussion

In this work, we demonstrated a DBCA framework using sample entropy to explore the complexity fingerprints at both voxel level and brain connectivity topology levels, which was made up of dynamical brain entropy mapping analysis and brain entropy based on complex network analysis. Taking the seafarer as an exemplar, the DBCA simultaneously generated the dynamical brain entropy maps and related connectivity topology exhibiting the qualitative complexity differences, which suggested the long-term seafarer’s experience really reshaped the brain functional network, and provided the new evidence about the occupational neuroplasticity.

### Local Activity Changes of Functional Neuroplasticity in Seafarers

The sliding window model has become a popular model for handling typical fMRI data (Allen et al., 2014). Studies have considered about the dynamic and non-linear changes of BOLD signal (Sokunbi, 2014; Niu et al., 2018; Zheng et al., 2020), which can be characterized by the complexity of the fMRI time series. As for us, under different window lengths, the brain complexity estimated by the sample entropy of seafarers and non-seafarers always showed significant results, indicating that the length of the sliding window is not sensitive to changes in brain complexity. Our result is estimated by computing brain entropy maps of entire subjects using a series of sliding windows.

Similar to the previous results of seafarers (Wang N. et al., 2018), the dynamic entropy values of the seafarer group in the cerebellum (Cerebelum_9_R/Cerebelum_8_R and Cerebelum_8_L) were significantly lower than those in the non-seafarer group, which is crucial for fine motor coordination. The findings indicated that the seafarers’ plasticity changes are stable and construct a regularity system in the cerebellum. Therefore, we can infer that the long-term offshore work has an obvious influence on the functional neuroplasticity of the human cerebellum, at least within the working life. However, it is not clear whether this change will disappear gradually after the seafarer retires or will accompany it for a lifetime.

On the other hand, in a relatively short period of time, the entropy value of the seafarer group in the fusiform gyrus (BA20) is higher than that of the non-seafarer group, which is also reflected in the previous brain entropy measurement (Wang N. et al., 2018). The fusiform gyrus (FG) is related to the ability of face processing (Ghuman et al., 2014); obviously, it is essential for social situations. Since the seafarer group has differential activation patterns in FG, we would like to infer seafarers suffering from social difficulties after experiencing a long voyage in isolating working conditions.

Interestingly, we did not find that the dynamic brain entropy results showed that the seafarer group had higher brain entropy value in the superior temporal gyrus and part of the frontal lobe (Rectus_L/Frontal_Sup_Orb), compared with the non-seafarer group. Sample entropy cannot show the same discrimination effect in dynamic evaluation and overall analysis. This difference can be attributed to its noise (i.e., physical motion), of which noise level at *N* = 90 (the data length of the dynamic analysis) is higher than *N* = 150 (the overall analysis of the data length) (Sokunbi, 2014). In our results, the peak intensity gradually decreased as the window length increases, while the noise level comes down.

### Topological Changes of Functional Neuroplasticity in Seafarers

Characterizing complex systems like the human brain can help in understanding its structure and function; especially with the advantage of rs-fMRI, it can meet the basic requirement for using complex network strategies that assess different brain areas over time (Onias et al., 2014). To date, most brain network studies have used linear correlations between brain regions to construct and interpret brain networks, like PCC. However, the PCC and linear correlation are only sensitive to the linear component and may ignore the non-linear relationship between brain regions. We are considering the so-called non-linear measurement that refers to a method that is sensitive to both linearity and non-linearity, such as sample entropy. Furthermore, complex network analysis can extract feature indicators in the brain network, and graph theory is an important tool for describing network features. Recently, merging evidence suggests that functional connectivity networks change dynamically over time (Hutchison et al., 2013; Hindriks et al., 2016). Specifically, the dynamic connectivity networks constructed from the original fMRI time series demonstrate less discrimination and less robustness in terms of topological measures such as small-worldness and efficiency for classification between the seafarers and the control group than the dynamic entropy-based connectivity networks (see Supplementary Figures 1–3 and Supplementary Table 1). In addition, the dynamical complexity changes are the focus of this study. Therefore, we estimated dynamic connectivity networks based on brain entropy map sequences, in order to detect the topological changes of functional neuroplasticity in seafarers.

We also find that the difference between seafarers and non-seafarers becomes more significant as the window length increases. This may be because the sample entropy is less than 85 at the time point, where the entropy value calculation ignores the detailed information in the BOLD signal (Chen et al., 2009); meanwhile, in the shorter-length data, the sample entropy is insufficient in distinguishing ability (Sokunbi, 2014).

In our results, the seafarer group demonstrates longer path lengths across all thresholds, the average path length *L*_*p*_ of the seafarer group increased significantly (*p* = 0.0296 < 0.05, *Len* = 90 TRs). Similarly, we note that the difference in path length is significant at *p* <0.05 above threshold *K* = 0.1, as more edges are retained (see Supplementary Table 2). In the small-world brain network, a shorter path length indicates a small amount of intermediate transmission in the integration pathway, thereby consolidating the accurate and rapid transmission of information in integrated neural communication (Kaiser and Hilgetag, 2006). On the contrary, a large number of intermediate transmissions will result in greater signal loss, signal distortion, and slower processing speed. Therefore, the study shows that the increased path length can be explained by the weaker processing speed of the seafarers’ brain network. Although there is no significant difference in the average clustering coefficient *C*_*p*_ between the seafarer group and the non-seafarer group (*p* = 0.2785 > 0.05, *Len* = 90 TRs), the average normalized clustering coefficient γ was significantly lower than that of the non-seafarer group (*p* = 0.0174 < 0.05, *Len* = 90 TRs). However, we rarely found the difference in clustering coefficient across all thresholds (see Supplementary Table 2), which would indicate that there are no between-group differences in the ability to “modularize” information processing. Under different window lengths, both the seafarer group and the non-seafarer group have small-world network attributes (*S* 1), which indicates that the small-world network is an inherent attribute of the human brain regardless of occupation.

With regard to the efficiency, the network efficiency of the same seafarer group is relatively low. The average global efficiency *E*_*glob*_ (*p* = 0.0005 < 0.05, *Len* = 90 TRs) and average local efficiency *E*_*loc*_ (*p* = 0.0009 < 0.05, *Len* = 90 TRs) is significantly different with the non-seafarer group. Interestingly, the Supplementary Table 2 results of each threshold have shown that the difference in global efficiency is significant above threshold *K* = 0.15, while the results of local efficiency are not statistically significant. Therefore, combined with the prior results, we could infer the seafarer might be weak in the long distance information procedure of each brain region, but the processing capability within regions is as efficient as the averages.

### Selection of Complexity Measures

Recently, many studies have shown that multi-scale entropy (MSE) (Costa et al., 2005) can measure the complexity of time series by taking into account different scales, where MSE is also proven to be effective in analyzing the rs-fMRI time series or physiologic data (Hu and Liang, 2012; Wang D. J. et al., 2018). However, the sample entropy of a time series corresponds to “scale 1” of the MSE analysis procedure (Costa et al., 2005; Sokunbi, 2014; Shafiei et al., 2019). Further, the aim of this study is to explore the dynamical complexity alteration caused by long-term occupational experience, which should be measured based on the continuous brain entropy time series. If the MSE with “scale 2” or “scale 3” is performed on original fMRI time series within the sliding windows, it generates the non-continuous brain entropy time series, which severely affects the decoding performance of the dynamical complexity of the brain. Thus, in this study, the sample entropy (i.e., MSE with scale 1) is chosen to estimate the entropy values in the proposed DBCA.

### Limitations and Future Direction

This study demonstrates the capability of DBCA in rs-fMRI capturing the differences between seafarers and non-seafarers, presumably activated activity, and functional connectivity alteration related to the occupational factors. However, these findings of DBCA in seafarers are needed to validate on large-scale datasets in our future work. It can be verified that the information theory-based methods make a contribution on brain functional networks, and further research is needed to explore the linear and non-linear interactions among multiple occupations in brain functional network analysis.

## Conclusion

In order to investigate the dynamical complexity fingerprints derived from the fMRI data, in this study, a novel DBCA framework was proposed to explore the dynamical complexity of brain activity and topological complexity changes of brain connectivity. Further, taking the professional seafarers as an exemplar, the complexity fingerprints related to occupational factors impacting functional neuroplasticity were explored, which validated the effectiveness of the proposed DBCA. On one hand, the dynamical brain entropy mapping analysis in DBCA showed that the seafarers with lower dynamical brain entropy values had stronger consistent brain activity in the cerebellum, which possibly contributed to the career performance in seafarers such as navigation skills, keeping balance, etc. On the other hand, based on the brain entropy-based complex network analysis, it was found that the occupational neuroplasticity was also supported by the complexity alteration of the functional connectivity of the seafarers, which likely meant that the processing network efficiency and modular processing capacity in seafarers were lower than those in the control group. In summary, the DBCA method is effective to explore the dynamic complexity changes at voxel-wise activity and region-wise connectivity, showing that occupational experience can reshape dynamic brain complexity fingerprints.

## Data Availability Statement

The datasets presented in this article are not readily available because our research project is still going on. Requests to access the datasets should be directed to NW (wangnizhuan1120@gmail.com).

## Ethics Statement

The studies involving human participants were reviewed and approved by the IRB of East China Normal University (ECNU). The patients/participants provided their written informed consent to participate in this study.

## Author Contributions

HY and HW: conceptualization, methodology, validation, formal analysis, writing – original draft, and funding acquisition. YC: software and writing – review and editing. YY, MX and JZ: conceptualization, investigation, and writing – review and editing. WZ: investigation, writing – review and editing, and data curation. CC and NW: conceptualization, resources, writing – review and editing, supervision, funding acquisition, and project administration. All authors contributed to the article and approved the submitted version.

## Conflict of Interest

The authors declare that the research was conducted in the absence of any commercial or financial relationships that could be construed as a potential conflict of interest.

## Publisher’s Note

All claims expressed in this article are solely those of the authors and do not necessarily represent those of their affiliated organizations, or those of the publisher, the editors and the reviewers. Any product that may be evaluated in this article, or claim that may be made by its manufacturer, is not guaranteed or endorsed by the publisher.

## Footnotes

1
http://rfmri.org/DPARSF

2
http://www.fil.ion.ucl.ac.uk/spm/software/

3
http://www.restfmri.net/forum/REST

4
https://people.cas.sc.edu/rorden/mricro/mricro.html
