# Supplementary material for: Dynamical Complexity Fingerprints of Occupation-Dependent Brain Functional Networks in Professional Seafarers
Source: Front Neurosci. 2022 Mar 18;16:830808. doi: 10.3389/fnins.2022.830808 (PMC8973415; doi:10.3389/fnins.2022.830808)
Supplement: Supplementary file 1 [file Data_Sheet_1.pdf]

Supplementary material for "Dynamical Complexity Fingerprints of Occupation-dependent Brain Functional Networks in Professional Seafarers"

Table S1 The mean of all network parameters for seafarers and non-seafarers at Len= 90TRs from original fMRI time series are presented, as well as the results of two-way ANOVA (*p*-value)

Properties	Parameters	Groups	Thresholds						
			0.05	0.1	0.15	0.2	0.25	0.3	p-value
		Seafarers	0.35	0.51	0.59	0.64	0.68	0.71	
		Non-	0.43	0.55	0.62	0.67	0.71	0.71	**0.0292**
		Seafarers	1.95	1.98	1.86	1.70	1.57	1.45	
		Non-	2.03	2.04	1.85	1.67	1.53	1.43	0.7479
		Seafarers	9.84	4.23	2.93	2.38	2.04	1.83	
Small-World		Non-	8.96	4.08	2.96	2.39	2.06	1.84	0.3281
		Seafarers	1.87	1.43	1.26	1.18	1.13	1.09	
		Non-	1.79	1.37	1.27	1.19	1.14	1.10	0.3982
		Seafarers	1.08	1.42	1.49	1.45	1.39	1.33	
		Non-	1.16	1.50	1.48	1.42	1.35	1.30	0.7638
		Seafarers	0.11	0.24	0.34	0.42	0.49	0.55	
		Non-	0.12	0.25	0.34	0.42	0.49	0.54	0.9712
Network Efficiency		Seafarers	0.40	0.59	0.68	0.74	0.78	0.82	
		Non-	0.40	0.58	0.67	0.74	0.78	0.81	0.0769

Table S2 The between-group p-values by two-sample T-test under different thresholds at Len= 90TRs are presented (p-value)

Properties	Parameters	Thresholds					
		0.05	0.1	0.15	0.2	0.25	0.3
		0.077	0.435	0.292	0.187	0.142	0.088
Small-World							
		0.134	0.13	0.096	0.136	0.181	0.249

	0.073	**0.035**	**0.024**	**0.019**	**0.022**	**0.016**
	0.34	0.26	0.2	0.181	0.087	**0.022**
$_{gl}$	0.129	0.087	**0.048**	**0.026**	**0.023**	**0.014**
Network Efficiency	0.085	0.229	0.169	0.204	0.197	0.263

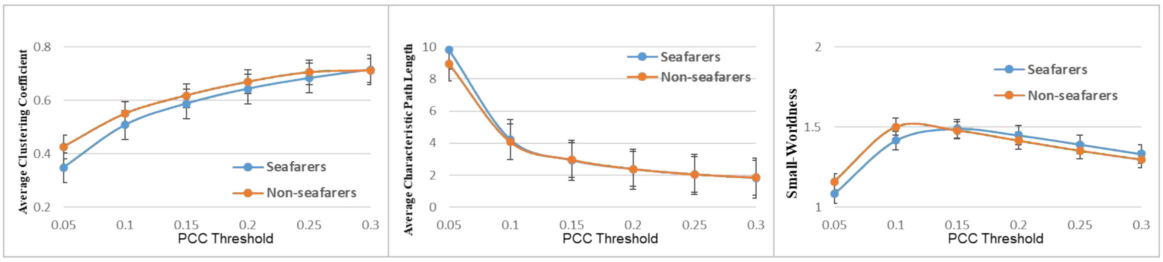

Figure S1 Average values of small world attributes and their parameters of the seafarers and non-seafarers based on original fMRI time series version under different thresholds at Len= 90TRs

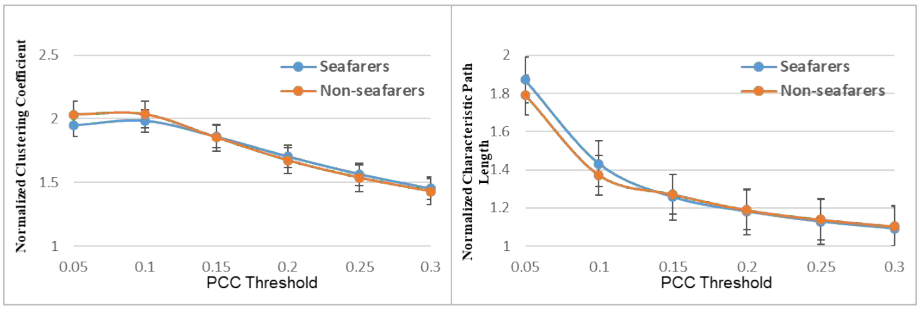

Figure S2 The average normalized path length and average normalized clustering coefficient of the seafarers and non-seafarers based on original fMRI time series under different thresholds at Len= 90TRs

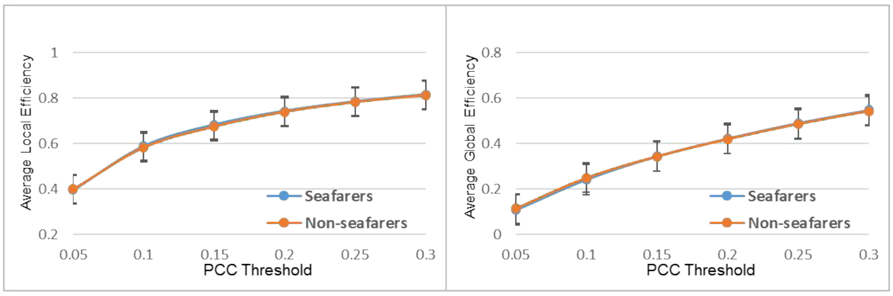

Figure S3 The average value of the efficiency attribute and its parameters of the seafarers and non-seafarers based on original fMRI time series under different thresholds at Len= 90TRs
